# The Latest Trend in Buttock Aesthetics: Brazilian Buttock Lift Reversal and Buttock Reduction

**DOI:** 10.1093/asjof/ojaf014

**Published:** 2025-03-04

**Authors:** Stanley A Okoro, Scarlet Lamb

## Abstract

**Background:**

The global popularity of Brazilian buttock lift (BBL) has led to a rise in the need for large volume fat transfer and disproportionate buttock and hip contouring. Becuase of the pandemic, there has been a rise in requests for BBL revision and buttock reduction.

**Objectives:**

The authors review their experience with a recent trend in BBL revision or buttock reduction to evaluate patient outcomes and complications.

**Methods:**

A retrospective chart review of all patients who underwent BBL revision or buttock reduction between February 2018 and December 2023 was conducted. Patient outcomes and complications were analyzed to assess the safety and outcome of performing these procedures.

**Results:**

A total of 123 patients were included in the study. Seventy-seven (63%) of these procedures were completed under local anesthesia with oral sedation. One hundred and eleven patients (90%) of patients undergoing BBL revision also included additional surgical procedures. The most common patient-reported dissatisfaction is residual adiposity in 10%, skin laxity in 6%, and persistent asymmetry in 4% in the study. The study revealed a 1% complication rate.

**Conclusions:**

BBL revision or buttock reduction is an increasingly popular body contouring procedure. It can be performed safely as an outpatient procedure by a well-trained and experienced plastic surgeon. The authors predict a significant rise in these procedures. In the author's experience, these procedures are associated with a very low rate of complications and a high level of patient satisfaction.

**Level of Evidence: 4:**

(Therapeutic)
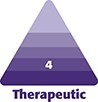

The Brazilian buttock lift (BBL) was one of the most popular plastic surgery procedures. The procedure's increased demand and popularity was mostly because of television, social media, influencers, and celebrities. The BBL reigned as a popular procedure despite being a high-risk surgery because of complications, such as fat embolism. However, it seems as though the BBL procedure's popularity is waning, and the pendulum has swung in the opposite direction.

After the pandemic of 2020, the authors noticed in their practice that there was an influx of plastic surgery patients presenting asking to get their BBLs reduced/reversed. Patients are complaining of excess fat placement contributing to poor shape as well as admitting to added weight since their initial BBL procedure. There is also a new social pressure to have a smaller buttocks and better contoured hips. Many celebrities have adapted a leaner shape. It appears that those responsible for the popularity of the BBL may be responsible for creating the demand for its revision. Patients are presenting to plastic surgeons requesting a smaller buttock, yet they still want to look good and have curves. The social pressure has created a new surgical problem that demands a modified surgical solution.

Currently, many plastic surgeons do not feel comfortable performing buttock reduction/revision with liposuction to the buttocks/hips. There is a fear of asymmetry, skin laxity, or leaving the patient with a “flat” buttock. It is important to discuss cutting-edge techniques that plastic surgeons can employ to meet the demands of their patient population. In this retrospective study, liposuction was most often combined with an energy-based device to provide optimal results. The authors reviewed their experience with the recent trend in BBL revision to evaluate patient outcomes and complications.

## METHODS

A retrospective chart review was performed of all patients undergoing buttock reduction performed by the senior author during a 6-year period (February 2018-December 2023). Patients with buttock implants or previous lower body lifts were excluded from the study. The analyzed data included patient demographics, surgical location, surgical specifications, simultaneous procedures, complications, and interventions. Simultaneous procedures are categorized as SmartLipo (Cynosure, Westford, MA), radiofrequency (RF) energy using BodyTite and Morpheus8, (InMode, Irvine, CA), RF energy using ThermiTight (Thermi, Austin, TX), skin excision, breast lift, and fat transfer to the hips. Initially, the senior author used laser-assisted liposuction as the sole skin-tightening adjunct procedure and then tried monopolar RF-assisted liposuction when it became available as a skin-tightening device. He noticed that the RF provided more uniform heating of the tissue and skin contraction. Later during the study period, the senior author acquired bipolar RF devices, which provided both internal and external heating of the tissue and skin. The author transitioned toward using only a bipolar RF device.

Complications were categorized as reported patient complaints discussed during postoperative visits: persistent asymmetry, skin laxity, and persistent localized adiposity.

This study was designed as a retrospective chart review without invasive procedures or interventions, and it solely involved anonymous, de-identified data collection, which does not require IRB approval according to our institution's research guidelines.

In accordance with the ethical principles set forth in the Declaration of Helsinki for research involving human participants, this study was conducted with utmost respect for participants’ rights, privacy, and welfare. All participants voluntarily provided informed consent, were informed of their rights to withdraw at any time without repercussions, and were assured that their data would be kept confidential and anonymous throughout the study process.

### Surgical Technique

**Figure 1.**
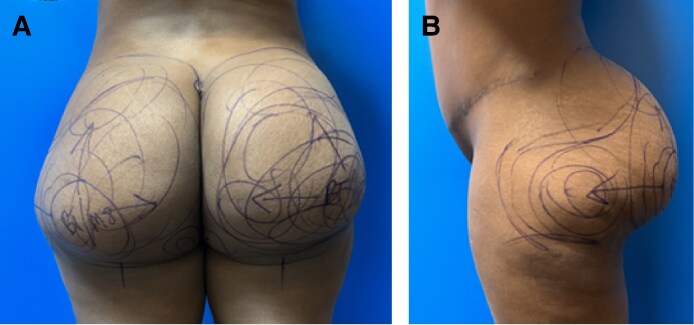
A 44-year-old female with preoperative markings. (A) The posterior view of the marking. (B) The lateral view of the marking. The circles on the image (A, B) indicate the areas of liposuction, and the arrow on the image indicates areas of bipolar radiofrequency energy.

All patients were assessed for medical risk before surgery. Indication for chemoprophylaxis of VTE/DVT was determined according to the 2005 Caprini risk assessment model. Written consent was provided, by which the patient agreed to the use and analysis of their data. All patients were marked in the preoperative holding area in a standing position (Figure 1). During preoperative marking, the patient indicated the areas of concern, and these areas were marked for fat reduction using a pinch test or skin tightening with an energy device.

Incisions were designed to be within the natural skin folds: the intergluteal fold and the bilateral infragluteal crease. In many instances, incisions from previous procedures were utilized. When general anesthesia was utilized, patients had a urinary catheter placed (when indicated) and had noninvasive blood pressure monitoring. Vital signs and urine output were continuously monitored and evaluated intraoperatively. Intraoperative fluid administration was given at the discretion of the surgeon and anesthesia provider based on vital hemodynamics and urine output. In cases where patients were placed under oral sedation, Bactrim DS, Hydrocodone-APAP, Promethazine, Alprazolam, and Gabapentin were administered by mouth at least 1 h before the start of the case. Patients undergoing general anesthesia received IV antibiotics (Cefazolin 2 g) at least 1 h before incision. Patients who are allergic to penicillin received either ciprofloxacin 400 mg IV or clindamycin 600 mg IV.

**Figure 2.**
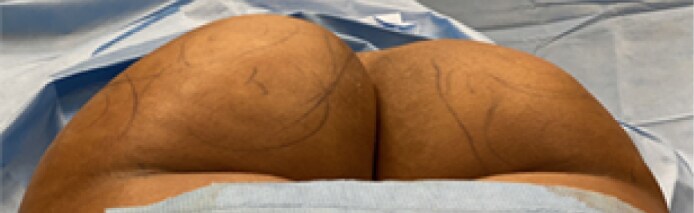
A 31-year-old female intraoperative photograph of immediate reduction of the right buttocks/hips.

Patients were placed in the prone position. All pressure points were protected. The tumescent solution consisted of 100 mL lidocaine 1% plain and epinephrine 1.5:1000/0.9% NaCl (1000 mL inj.). Two hundred milliliters of sodium bicarbonate are added to the tumescent solution of the patient who is under oral sedation. Solutions were warmed to 40 °C with Medical Warming Cabinet. Power-assisted liposuction was always performed. Infiltration and liposuction were performed in the deep and superficial subcutaneous layers. Liposuction was employed with a 4 mm spiral tip, a 5 mm balloon tip, and a 4 mm DelVecchio tip. Determination of the surgical endpoint was made with a “pinch test” (3-4 cm), evaluation of the amount of fat removed, and visual inspection of symmetry (Figure 2).

Where there is concern for skin laxity preoperatively, energy-based devices were employed on a case-by-case basis. SmartLipo Triplex (laser; Cynosure, Westford, MA) was utilized in the deep subcutaneous space. ThermiTight is set to an internal target temperature of 70 °C, and the probe was utilized in the deep subcutaneous space. Surface skin temperature was monitored with FLIR camera. BodyTite is set to an internal target temperature of 70 °C with an external target temperature of 38 °C, and the probe was utilized in the deep subcutaneous space (Video) Morpheus8 Body settings, as described in Table 1.

**Table 1.** Morpheus8 Body Settings for All Skin Types

Mode	Depth	Energy	Pulse per second
Cycle	7 mm	40	1.0
Burst	7, 5, 3 mm	30	1.0

Before completion of the case, liposomal bupivacaine (Exparel, Pacira BioSciences, Inc, Brisbane, CA) was infiltrated locally for long-acting pain control. Incisions were left open for drainage. Dressings included absorbent pads and a compression garment.

All cases were performed in an outpatient facility accredited by Quad A (formerly the American Association for Accreditation of Ambulatory Surgery Facilities). Postoperatively, patients were observed in the postanesthesia care unit (PACU). Patients were discharged to home once appropriate PACU criteria were met. Patients were followed up to 7 days postoperatively and as long as 1 year postsurgery. Our practice has evolved to comprise early postoperative ambulation, compression garments (Marena, Lawrenceville, GA), preoperative and postoperative lymphatic drainage (BalancerPro, Beverly Hills, CA), and postoperative lymphatic massages (Smoothshapes, Cynosure, Westford, MA).

## RESULTS

**Figure 3.**
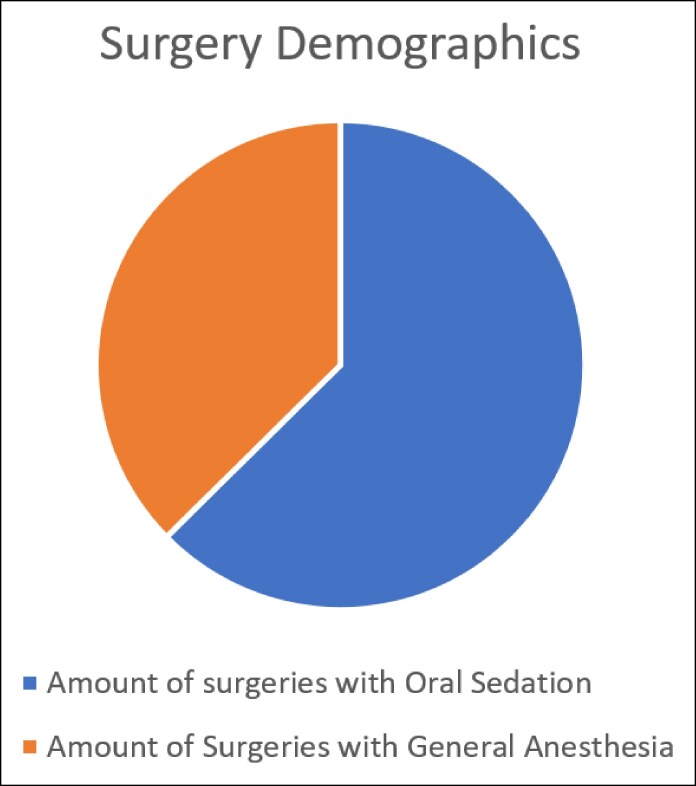
Buttocks reduction surgical demographics among patients who received oral sedation protocol compared with general anesthesia.

A total of 123 patients underwent buttock reduction with liposuction to the buttocks/hips. Patients’ ages ranged from 20 to 59 with a mean age of 35. Females comprised 96.75% of the cohort, and 3.25% were males. Seventy-seven patients (63%) had their procedure performed under oral sedation and 46 (37%) by general anesthesia (Figure 3). The average amount of tumescent infiltrated into the left buttock/hip was 941.1 cc. The average amount of tumescent infiltrated to the right buttock/hip was 1022.2 cc. The average amount of tumescent in total was 1861.7 cc (Supplemental Figure 1). The average amount of fat removed from the left buttock/hip was 706.5 cc. The average amount of fat removed from the right buttock/hip was 740.9 cc. There was no significant difference in the amount of tumescent fluid used or lipoaspirate between the left and the right buttock/hip in most patients. The average amount of fat removed from bilateral buttock/hip was 1433.4 cc (Supplemental Figure 2). The average amount of time for the procedure was 136.4 min.

**Figure 4.**
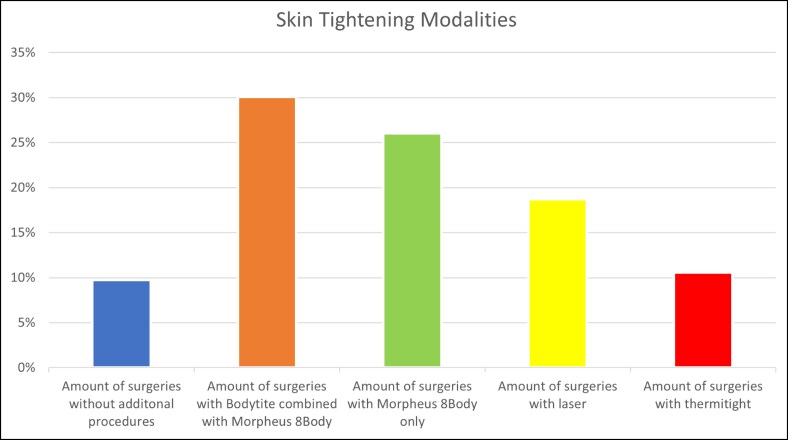
A comparison between skin-tightening modalities utilized during liposuction of the buttocks and hips.

Ninety percent (90.2%) of the cohort had a simultaneous procedure with liposuction to the buttock/hips. A skin-tightening procedure for skin laxity was performed in 105 patients (85.3%). Of the energy-based devices incorporated at the time of surgery, 30% of the cohort had BodyTite with Morpheus8 Body, 18.7% of the cohort had SmartLipo laser, 10.6% had ThermiTight, and 26.0% had Morpheus8 Body Microneedling (Figure 4). The follow-up period ranged from 3 to 24 months. The average length of follow-up is 12 months.

**Figure 5.**
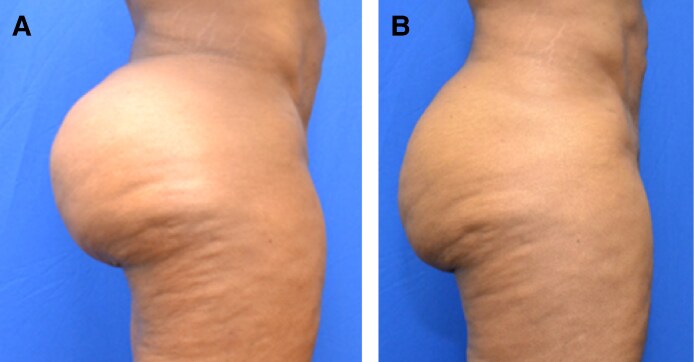
A 34-year-old female (A) before and (B) 12 months after liposuction to the buttocks and hips with SmartLipo laser skin tightening.

**Figure 6.**
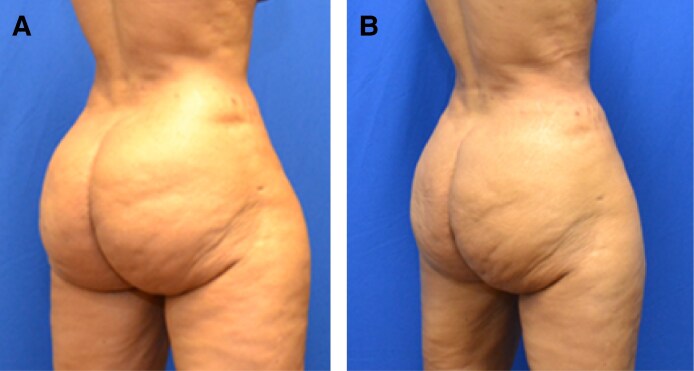
A 61-year-old female (A) before and (B) 3 years after liposuction to the buttocks and hips.

**Figure 7.**
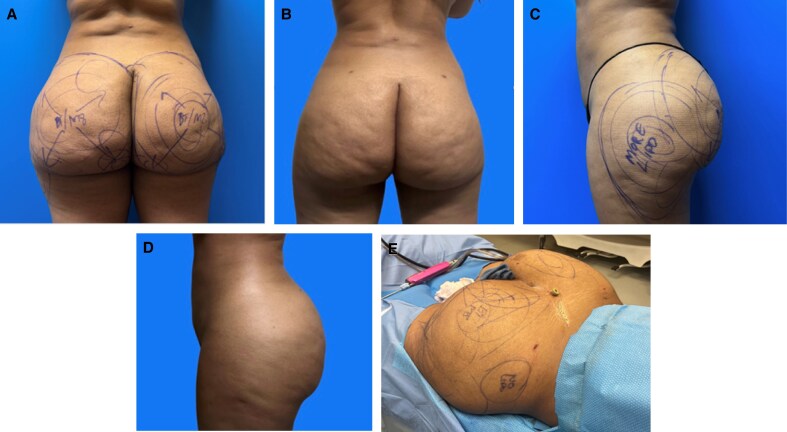
A 30-year-old female 10 months after liposuction to the buttocks and hips (right 600 cc and left 850 cc) with radiofrequency skin tightening (35 kJ per buttock/hip) and radiofrequency microneedling. (A, C) The preoperative marking where the circles indicate areas of liposuction and the arrows indicate areas of BodyTite and Morpheus8 Microneedling (M8). (B, D) The postoperative results. (E) The intraoperative photograph of the same patient.

Patient satisfaction is discussed during postoperative appointments. Seventy-five percent (75.7%) of the cohort was satisfied with their postoperative outcome (Figures 5-7, Supplemental Figures 3-5). Complaints after the procedure included residual adipose tissue in 10.6%, residual skin laxity in 6.5%, and asymmetry in 6.5% of the cohort (Supplemental Figure 6). One patient experienced opening of her previously closed liposuction incision, which we considered a wound dehiscence.

Fifteen patients, or 12% of the cohort, returned to our practice for additional liposuction and/or skin tightening of the buttocks/hips.

## DISCUSSION

**Figure 8.**
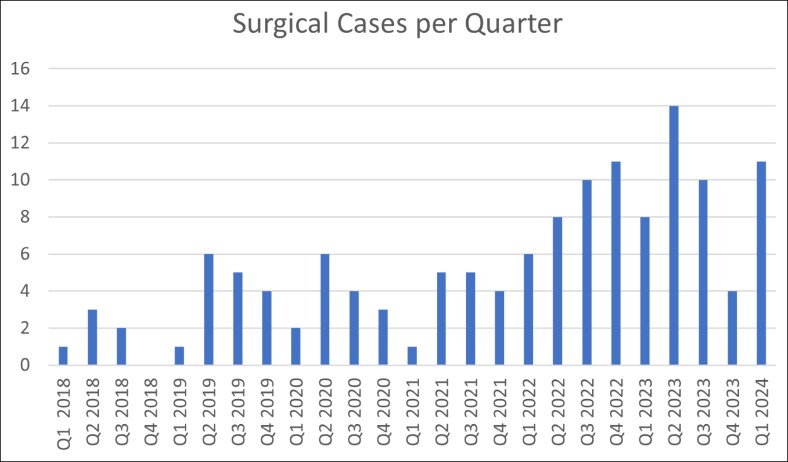
Understanding the trends of plastic surgery patients based on amount of Brazilian buttock lift revision procedures performed each quarter.

The popularity of BBL reversal, BBL reduction, and buttock reduction procedures will continue to increase in 2024 and beyond, secondary to social trends.^1^ Based on the trend in our practice (Figure 8), we predict that more and more practices will start offering BBL reductions. To stay competitive and keep patients in their own practice, plastic surgeons need to learn how to confidently offer BBL revision or reversal for this new surgical problem.

Patients are presenting with new problems related to their buttocks/hips: (1) excess fat, (2) skin laxity, and (3) skin excess. Although many surgeons feel comfortable with treating excess fat with liposuction, we must address all the problems the patient complains about in consultation. Addressing skin laxity and skin excess can be accomplished with the skin-tightening modality offered by the practice. The efficacy and safety of RF in skin laxity have been proven by many studies.^2,3^ In our practice, RF-based devices offered the most favorable results.

### Limitations

This study may have been affected by single surgeon experience, short follow-up (3 months in some cases), heterogeneity of energy devices used, limited to nonexcisional techniques, limited to reversal of BBL by fat transfer only, and the selection bias because of its retrospective nature. Additionally, aesthetic outcomes are subjective.

## CONCLUSIONS

With the immense popularity of the BBL procedure performed by cosmetic and plastic surgeons, it was only a matter of time before plastic surgeons were called upon for “BBL reversal.” An understanding of liposuction techniques in combination with skin tightening can allow for the most optimal aesthetic result. We predict a significant rise in these procedures with increasing age and weight gain. In the author's experience, these procedures are associated with a very low rate of complications and a high level of patient satisfaction. Larger prospective studies will be informative on the best practices for patient satisfaction and prevention of complications in this population of patients.

## Supplementary Material

ojaf014_Supplementary_Data
