# Supplementary material for: The Latest Trend in Buttock Aesthetics: Brazilian Buttock Lift Reversal and Buttock Reduction
Source: Aesthet Surg J Open Forum. 2025 Mar 4;7:ojaf014. doi: 10.1093/asjof/ojaf014 (PMC12209810; doi:10.1093/asjof/ojaf014)

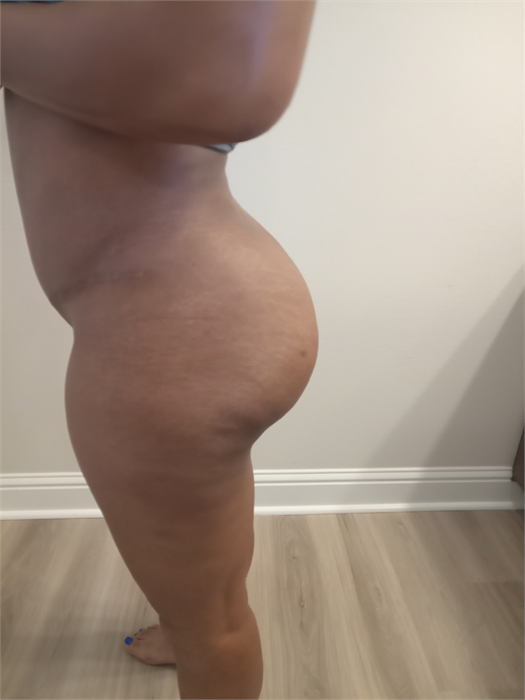
Supplementary Figure 3: 42-year-old female 12 months after liposuction to the buttocks and hips with Radiofrequency skin tightening and Radiofrequency microneedling


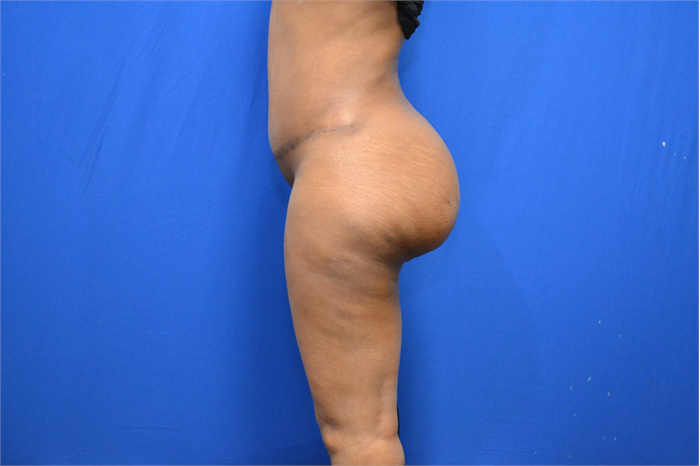

Supplement: ojaf014_Supplementary_Data [file ojaf014_Supplementary_Data.zip › Supplementary Figure 3.docx]
