# Supplementary material for: The Latest Trend in Buttock Aesthetics: Brazilian Buttock Lift Reversal and Buttock Reduction
Source: Aesthet Surg J Open Forum. 2025 Mar 4;7:ojaf014. doi: 10.1093/asjof/ojaf014 (PMC12209810; doi:10.1093/asjof/ojaf014)

Supplementary Figure 4: 24-year-old female 6 months after liposuction of the buttocks and hips with radiofrequency microneedling


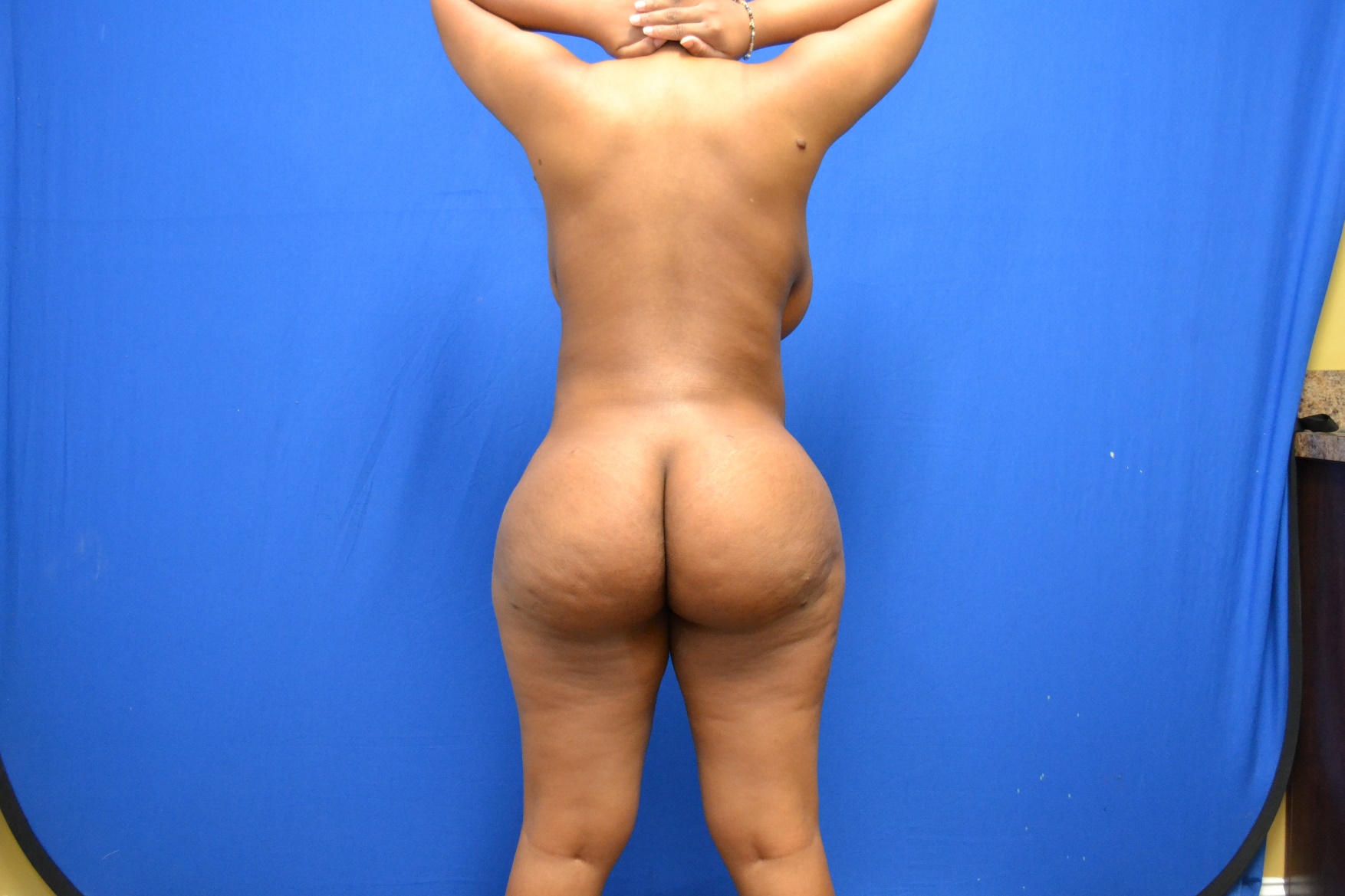

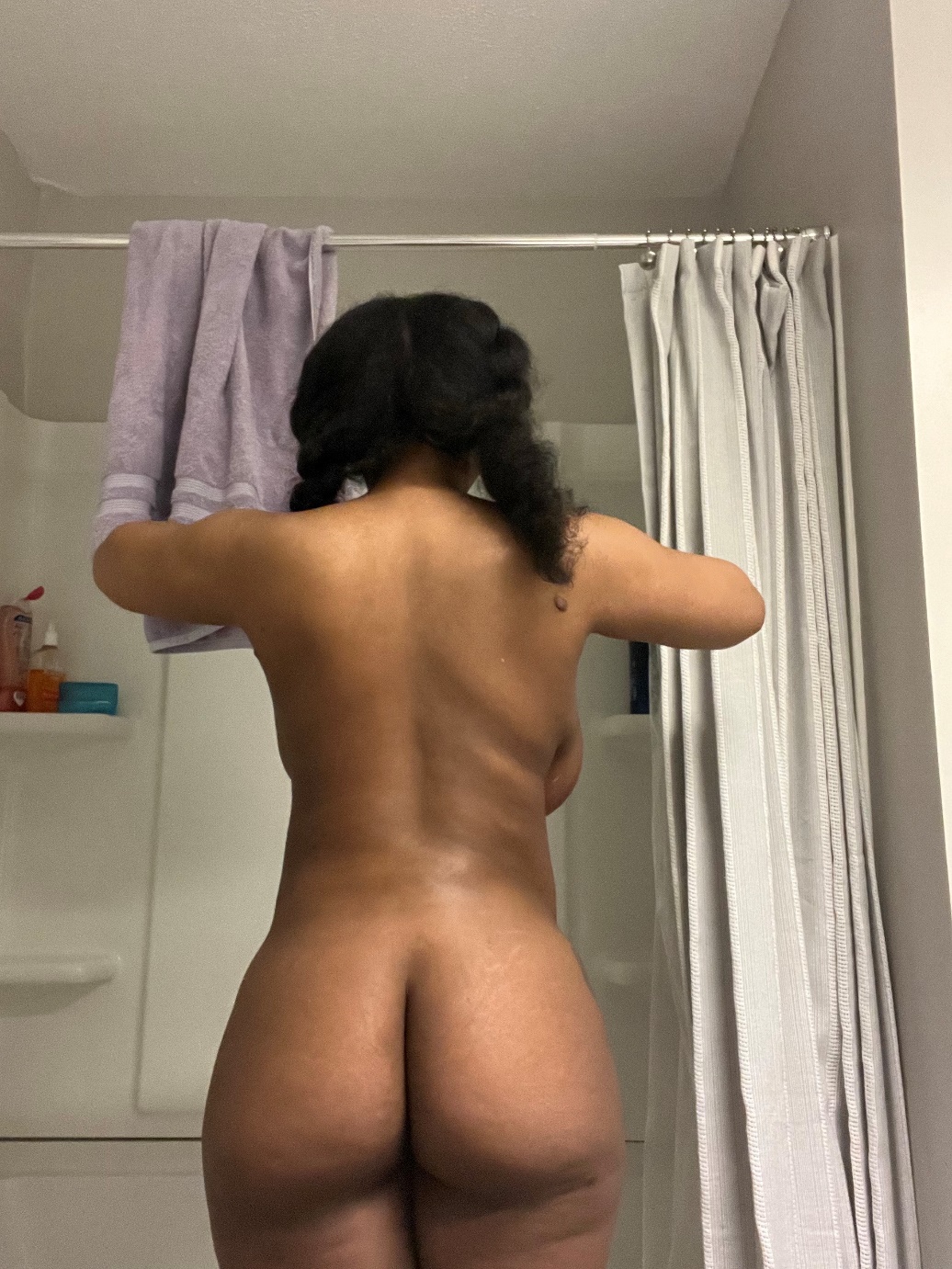

Supplement: ojaf014_Supplementary_Data [file ojaf014_Supplementary_Data.zip › Supplementary Figure 4.docx]
